# Single-Session Interventions To Enhance HIV Outcomes among Adolescents and Young Adults: A Systematic Scoping Review and Implications for Integrating HIV and Mental Health Services

**DOI:** 10.1007/s10461-025-04834-4

**Published:** 2025-08-01

**Authors:** Juan Pablo Zapata, Andy Rapoport, Annie Wescott, Shivranjani Gandhi, Tyra Cole Bergstrom, Andrés Alvarado Avila, Lisa M. Kuhns, Robert Garofalo, Jessica Lee Schleider

**Affiliations:** 1 Department of Medical Social Sciences, Feinberg School of Medicine, Northwestern University, Chicago, IL USA; 2 Institute for Sexual and Gender Minority Health and Wellbeing, Feinberg School of Medicine, Northwestern University, Chicago, IL USA; 3 Galter Health Sciences Library, Feinberg School of Medicine, Northwestern University, Chicago, IL USA; 4 Department of Pediatrics, Feinberg School of Medicine, Northwestern University, Chicago, IL USA; 5 Division of Adolescent and Young Adult Medicine, Anne & Robert H. Lurie Children’s Hospital of Chicago, Chicago, IL USA; 6 Department of Psychology, Northwestern University, Evanston, IL USA

**Keywords:** Single session interventions, Adolescent and young adult, HIV treatment, HIV prevention, Mental health outcomes

## Abstract

Significant progress has been made in HIV prevention and treatment for adolescents and young adults (AYA), but integrating mental healthcare remains essential to address risks and improve treatment engagement. Single-session interventions (SSIs) offer a cost-effective, scalable way to improve access to HIV and mental health services. While evidence shows SSIs reduce sexual risk behaviors and enhance mental health, their combined impact on both areas has not yet been reviewed. This scoping review evaluates SSIs designed for AYA that target HIV treatment and prevention outcomes, while assessing whether these SSIs had any secondary effects on co-occurring mental health outcomes. A systematic search of databases through August 2024 identified 21 SSIs that included HIV-related outcomes, such as intentions to test, improvements in HIV knowledge, attitudes toward HIV, PrEP uptake, and treatment engagement, and any reported mental health outcomes. Of the 21 studies analyzed, 19 focused on HIV prevention, showing significant improvements in knowledge, reduced stigma, and better self-assessment of HIV risk. The remaining two studies examined treatment-related outcomes, with some interventions like video-based programs and motivational interviews improving treatment initiation and attitudes toward adherence among AYA. Notably, only two of the 21 studies explicitly integrated mental health-related outcomes, despite nearly all interventions targeting change mechanisms common to mental health-focused interventions. Findings from this review highlight the promise of SSIs for improving HIV-related outcomes among AYA. However, to maximize their impact, future research should incorporate mental health-related outcomes into these interventions.

**Supplementary Information:**

The online version contains supplementary material available at 10.1007/s10461-025-04834-4.

## Resumen

Se han logrado avances importantes en la prevención y tratamiento del VIH entre adolescentes y adultos jóvenes (AAJ); sin embargo, la integración de la salud mental sigue siendo esencial para reducir riesgos y mejorar la adherencia al tratamiento. Las intervenciones de una sola sesión (SSIs, por sus siglas en inglés) ofrecen una forma rentable y escalable de ampliar el acceso a servicios de VIH y salud mental. Aunque existe evidencia de que las SSIs reducen conductas sexuales de riesgo y mejoran el bienestar mental, su impacto conjunto en ambas áreas no ha sido sistemáticamente evaluado. Esta revisión de alcance analiza SSIs dirigidas a AAJ que abordan resultados relacionados con el VIH, evaluando también si tuvieron efectos secundarios sobre la salud mental. Se realizó una búsqueda sistemática en bases de datos hasta agosto de 2024, identificando 21 SSIs que incluyeron resultados vinculados al VIH, como intención de realizar pruebas, conocimiento, actitudes, uso de PrEP y compromiso con el tratamiento y cualquier resultado reportado sobre salud mental. De los 21 estudios, 19 se enfocaron en prevención, mostrando mejoras en conocimientos, menor estigma y mejor autoevaluación del riesgo. Dos estudios evaluaron resultados de tratamiento, observando que algunas intervenciones, como programas en video y entrevistas motivacionales, favorecieron el inicio del tratamiento y actitudes hacia la adherencia. Solo dos estudios integraron explícitamente resultados relacionados con salud mental, a pesar de que la mayoría apuntaban a mecanismos de cambio comunes con intervenciones psicológicas. Los hallazgos destacan el potencial de las SSIs para mejorar resultados relacionados con el VIH, recomendando mayor integración de componentes de salud mental en futuras investigaciones.

## Introduction

Despite significant advancements in HIV treatment through antiretroviral therapy (ART) and the development of biomedical prevention strategies like treatment as prevention and oral and injectable pre-exposure prophylaxis (PrEP) [1], HIV remains one of the leading causes of death among youth ages 13 to 24, or adolescents and young adults (AYA), worldwide [2]. This group experiences the highest rates of dropout and loss to follow-up across the treatment continuum, which includes HIV testing, diagnosis, linkage and retention in care, ART adherence, and viral suppression [3–6]. Compared to younger children (ages 0 to 13) and older adults (ages 25 and above), AYA are the least likely to be aware of their HIV status and achieve viral suppression, while also having the highest incidence of new HIV infections [7].

### The Challenge of Integrating AYA HIV Care and Mental Health Support

Over the past several decades, attention to the intertwined epidemics of HIV and mental health among AYA has increased. This is because higher HIV risk and lower care engagement often coincide with mental health challenges [8–12]. The relationship between mental health issues and HIV is complex and bidirectional. Individuals living with HIV are susceptible to AIDS-related opportunistic infections, which can lead to, among other things, cognitive and neurological damage [13]. Additionally, mental health problems may arise as a side effect of ART [14] or from the stigma and stress tied to the infection and treatment process [15]. Similarly, among HIV-negative individuals, depression and substance use disorders, which often co-occur are known to increase the risk of behaviors that facilitate HIV transmission, such as risky sexual activity [16] and injection drug use [17].

Improving the health of AYA requires holistic interventions that address mental health and HIV care simultaneously. Unfortunately, many current approaches focus narrowly on either HIV-specific outcomes or mental health issues, neglecting the interconnectedness of the two [18]. Research underscores the urgent need for universal mental health screenings and the integration of mental health treatment into routine HIV care [11]. Indeed, integrating mental health services into HIV care offers significant benefits [19], including better clinical outcomes for both HIV and mental health conditions, reduced substance use, decreased stigma, improved social functioning, and higher patient engagement in care [20–22]. Despite this clear need and benefit, most AYAs living with or at risk for HIV do not receive any mental health care at all, due in part to global provider shortages and fragmented service delivery systems [11, 22, 23]. Moreover, existing HIV programs fail to include mental health support or screenings; the few HIV programs that do incorporate mental health interventions are often limited to research evaluations and rarely implemented on a larger scale [19]. Furthermore, existing HIV interventions with mental health components are typically multi-session and require trained mental health specialists, making them difficult to implement in routine care, especially in resource-limited areas [23]. Addressing these barriers is essential to ensure mental health support becomes an integral, scalable part of HIV-related care.

### Harnessing Single-Session Interventions: Evidence and Opportunities for Advancing HIV and Mental Health

Single-session interventions (SSIs) present a promising means of addressing these challenges. SSIs are defined as “structured programs that intentionally involve only one visit or encounter with a clinic, provider, or program” [24]. These interventions can be delivered by trained providers or through digital, self-guided formats. They are utilized in a variety of settings, including specialty mental health clinic waitlists, emergency departments, primary care, schools, and even smartphones. A recent umbrella review synthesizing findings from over 400 clinical trials revealed that 83.33% of the reviews (20 out of 24) reported significant and clinically meaningful effects for at least one outcome, such as reductions in depression or anxiety, or improvements in treatment engagement or uptake [25]. Though less extensively studied, SSIs have also shown potential as a prevention strategy for mitigating sexual risk. A meta-analysis of 67 clinical trials found that, compared to controls, SSIs significantly reduced sexual risk behaviors, such as frequency of unprotected sex and increased condom use, among AYA (Cohen’s *d* = 0.19) [26]. Notably, outcomes from both reviews were observed across various time points, in some cases lasting up to 12 months.

How can brief interventions produce *any* positive change across both mental health problems and sexual risk behaviors, let al.one benefits lasting months post-intervention? SSIs are grounded in intervention theories of change, which target theory-driven mechanisms underlying sustained shifts in beliefs, emotions, and behaviors [27]. Evidence points to overlap in the theoretical frameworks that underpin SSIs addressing both mental health [24] and sexual risk behaviors [26], as well as frameworks cited in effective interventions for HIV care [28]. Although no SSIs were identified in the reviews as addressing both mental health and sexual risk outcomes simultaneously, their shared mechanisms of change suggest the potential for such dual-impact interventions. For example, SSIs based on social cognitive theory often include components aimed at enhancing participants’ motivation for safer sex practices (e.g., through self-reflection and goal setting) [29] or promoting behavioral activation to reduce depressive symptoms [30]. Their shared theoretical underpinnings suggest that these interventions could generate synergistic effects, improving outcomes across both domains. This potential synergy is particularly relevant for addressing the intertwined challenges of HIV and mental health.

Despite these successes in mental health and sexual risk prevention, no existing reviews have explored SSIs that simultaneously target both outcomes within a single intervention. Furthermore, current reviews of SSIs addressing sexual risk behaviors focus primarily on prevention strategies, such as condom promotion and risk reduction, with limited investigation into their potential for improving HIV care outcomes among AYA living with HIV, such as ART adherence or viral suppression. Additionally, many reviews narrow their scope to specific measures, such as condomless anal sex, while excluding other critical HIV prevention strategies, such as PrEP. Interestingly, while several of the SSIs included in these reviews were adapted from mental health approaches, such as motivational interviewing, and targeted common mechanisms of change prevalent in mental health interventions, they did not include mental health-related outcomes. A systematic understanding of the existing research is critical to formalizing efforts to leverage SSIs as a tool for streamlined, integrated services addressing both HIV and mental health outcomes. Further research is needed to evaluate their full potential and optimize their use in diverse populations.

### Present Study

This scoping review has two primary objectives: (1) to evaluate SSIs designed for AYA that target HIV treatment and prevention outcomes and (2) to assess the extent to which these interventions also address mental health outcomes. Our methodology consists of two key steps. First, we will systematically review SSIs targeting HIV outcomes to determine how many also report mental health outcomes. Second, we will revisit two recent systematic reviews [25, 31] of SSIs among AYA to identify studies where mental health outcomes were the primary focus and HIV outcomes were secondary, potentially leading to the exclusion of HIV-related results from these reviews. By combining these approaches, we aim to capture all relevant studies, regardless of whether these outcomes are listed as primary or secondary, delivering a more holistic understanding of SSIs’ possible dual impact.

## Method

### Search Strategy

This review followed the PRISMA Extension for Scoping Reviews (PRISMA-ScR) guidelines, as detailed in Supplementary File 1 [32]. Methods and goals for this scoping review were pre-registered in Open Science Framework (OSF) [33] before initiating search procedures. The review authors partnered with a research librarian (ABW) to create a comprehensive search of the literature. The following databases were searched on 26 June 2024: Ovid MEDLINE, Cochrane Library (Wiley), APA PsycINFO (Ebsco), Web of Science (Clarivate), and ProQuest Dissertation and Theses Global. The search strategy included a combination of controlled vocabulary and keyword searching related to brief interventions for adolescents with HIV. The full search strategy is detailed in the supplementary materials (Supplementary File 2) All databases were searched from inception to present without the use of filters or limits. Records were downloaded and underwent multi-pass deduplication in a citation management software (EndNote) and unique records were uploaded to a screening platform (Rayyan) for initial screening by two independent reviewers. Additionally, a review of articles captured by our lab in past meta-analysis & umbrella review was conducted.

### Inclusion and Exclusion Criteria

Studies included in this review met the following criteria: (1) published in English, (2) assessed the effects or associations of a single-session intervention on HIV prevention (including HIV testing and/or condom use for vaginal, anal, or oral sex), ART adherence, retention in care, or related biomedical outcomes (e.g., viral suppression, CD4 count), (3) reported quantitative outcomes, (4) focused on or included samples of AYA aged 13–24, and (5) were published between 1990 and June 4, 2024.

Exclusion criteria were as follows: (1) studies that did not focus on the specified age group or failed to provide disaggregated data for individuals aged 13–24, (2) studies that did not examine a single-session intervention, characterized as a structured program involving a visit or encounter with a clinic, provider, or program [25, 31], (3) works that were not full peer-reviewed articles presenting original empirical analyses (e.g., editorials, commentaries, reviews, abstracts), (4) non-experimental/intervention studies targeting motivation to engage with HIV prevention and treatment services across the HIV care continuum, (5) single-session interventions focused exclusively on the provision of pharmacotherapy (e.g., ART-only programs), and (6) systematic reviews, literature summaries, opinion pieces, or protocol papers. Initial study selection was conducted by four members of the author team (JPZ, AR, AA, AG) in collaboration with the research librarian (ABW). Disagreements were resolved via discussion.

### Data Extraction

Previous systematic reviews [26, 34] have demonstrated the effectiveness of SSIs and brief interventions in reducing sexual risk behaviors, particularly in areas such as unprotected sex and condom use. Building on this foundation, the current review shifts its attention to SSIs designed to address HIV-related outcomes, specifically, which have not been thoroughly examined in prior reviews. To address this gap, we systematically extracted and coded data on the following outcomes common to studies of HIV prevention and treatment programs: intentions to undergo HIV testing, actual HIV testing behavior, improvements in HIV education, changes in sexual practices, attitudes toward HIV, perceived HIV risk, communication with primary or casual partners about HIV status, PrEP usage and adherence, ART adherence, and attitudes toward sustaining ART adherence. Additionally, we coded various study characteristics, including minimum and maximum participant ages based on inclusion criteria, sample sizes, study and intervention locations, study designs, the format of the SSIs, and the extent of provider involvement. We also documented where in the care continuum the SSI was implemented and whether the intervention was conducted in a group or individual setting. Furthermore, we captured details about any theoretical frameworks used in the intervention design, the intervention’s duration, follow-up periods, types of comparators or controls, and any outcomes related to mental health, referred to as psychosocial factors. All included studies were double-coded by two members of the author team, with subsequent meeting to establish consensus, and all remaining misalignments and proposed finalized codes were reviewed by the first author.

## Results

### Study Selection and Inclusion

**Fig. 1:**
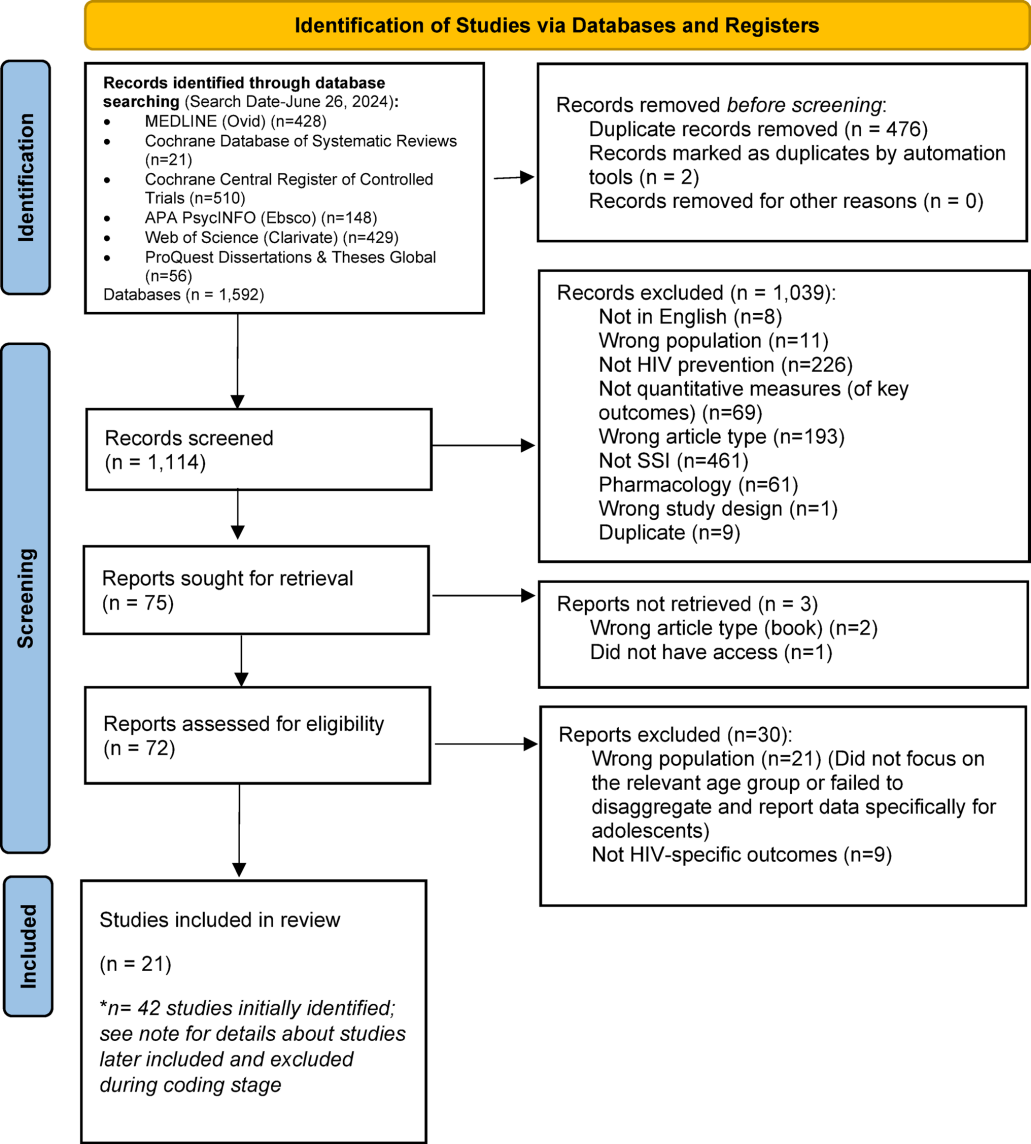
PRISMA Diagram. *n* = 15 studies were excluded for not focusing on the relevant age group or failing to disaggregate and report data specifically for AYA. *n* = 5 studies were excluded because the intervention was not a single session. *n* = 1 study was excluded due to inaccessibility, *n* = 1 was excluded as it was a duplicate, *n* = 1 was excluded for lacking an HIV primary outcome, and *n* = 1 was excluded because it was a dissertation. *n* = 1 study was added as a newly identified study, *n* = 1 was included via a review of prior lab meta-analyses and umbrella reviews, and *n* = 1 was added as a follow-up study building upon the previously excluded dissertation

The scoping review methodology is shown in Fig. 1 (PRISMA Chart). A total of 21 articles were ultimately included in the review [35–55]. The chart details each stage of the selection process, highlighting the points where articles were excluded as well as where additional studies were incorporated into the final analysis.

### Characteristics of Included Studies

As outlined in Table 1, the studies were published between 1992 and 2024 (*M* = 2010; *SD* = 9), with 33.3% being published in the last 5 years (2019 or later) and 38% in the last decade (since 2014). The average minimum and maximum ages of participants in the interventions were 15.23 and 21.05 years, respectively (excluding four studies [44, 49–51] that only provided age ranges). The majority of the interventions were conducted in the United States (*n* = 15), while the remaining studies took place in other countries, including Spain (*n* = 1), Zambia (*n* = 1), Hong Kong (*n* = 1), Turkey (*n* = 1), India (*n* = 1), and Saudi Arabia (*n* = 1). Among the analyzed studies, four focused on at least one of the WHO-designated key populations prioritized for HIV prevention, diagnosis, treatment, and care. These key populations, often recruited within the same study, included men who have sex with men (*n* = 3), transgender and gender-diverse individuals (*n* = 2), and people who inject drugs (*n* = 2). Regarding the control groups, three studies did not include a control group. Among the remaining 18 studies, the control group conditions varied: other non-active comparator (*n* = 9), psychoeducational (*n* = 3), no-treatment (*n* = 3), waitlist (*n* = 1), historical comparison (*n* = 1), and usual care (*n* = 1). Of the 12 studies that used either an “other non-active comparator” or “psychoeducational” control, five included control content unrelated to HIV or mental health, three focused on social and/or emotional health, and four were partially related to HIV.

**Table 1:** Reviewed articles

Article	Min Age	Max Age	Sample Size	Study Location	Study Design Type	Location of Intervention	Format of SSI Represented	Provider involvement in SSI	Group vs. Individual-based SSI	Place in Care Continuum
Abdullah et al. (2005)	18	25	154	Hong Kong	Pre-Post Assessment	Police Training School	Digital, In-Person (Hybrid)	Yes	Group-based	Prevention
Abolfotouh (1995)	14	19	838	Saudi Arabia	Other	Male Secondary School	In-Person	Yes	Group-based	Prevention
Agha and Van Rossem (2004)	14	23	416	Zambia	RCT	Secondary Boarding Schools	In-Person	Yes	Group-based	Prevention
Ashworth et al. (1992)	14	18	1194	United States	Cohort Study	High Schools	In-Person	Yes	Group-based	Prevention
Ballester-Arnal et al. (2017)	18	25	467	Spain	Pre-Post Assessment	College Campus	In-Person	Yes	Group-based	Prevention
Bangi et al. (2013)	14	21	264	United States	RCT	Community Based Organization	In Person	Yes	Group-based	Prevention
Brus and Jennit (2009)	15	18	1168	India	Pre-Post Assessment	High Schools	In Person	Yes	Group-based	Prevention
Ergene et al. (2005)	18	21	387	Turkey	Pre-Post Assessment	Other	In Person	Yes	Group-based	Prevention
Harper et al. (2009)	12	21	378	United States	Pre-Post Assessment	Community-Based Organization	In Person	Yes	Group-based	Prevention
Jenner et al. (2023)	18	19	1770	United States	RCT	Community-Based Clinic	Video format	Yes	Group-based	Prevention
Kamke et al. (2020)	12	19	47	United States	Other	Other	Web-based	None	Individual-based	Prevention
Karnik et al. (2023)	16	25	329	United States	RCT	Community-Based Clinic	Web-based	None	Individual-based	Prevention
Kennedy et al. (2013)	18	24	136	United States	Pre-Post Assessment	Community-Based Organization	In-person, human-guided	Yes	Group-based	Prevention
Neumann et al. (2018)			4,003	United States	Other	Specialty Clinic	In Person, Video	None	Group-based	Achievement and Maintenance of Viral Suppression
O’Grady et al. (2009)			108	United States	Pre-Post Assessment	Other	In Person, Video	Yes	Group-based	Prevention
Roye et al. (2007)	15	21	400	United States	RCT	Community-Based Clinic	In Person, Video	Yes	Individual-based	Prevention
Widman et al. (2020)	15	16	226	United States	RCT	Other (High School)	Web-based	None	Individual-based	Prevention
Yeagley et al. (2012)	13	24	18	United States	Pre-Post Assessment	Other (University Clinic)	In Person	Yes	Individual-based	Achievement and Maintenance of Viral Suppression
Jemmott et al. (1992)			157	United States	Other (Randomized Field Trial)	Other (High School)	In Person	Yes	Group-based	Prevention
McCrimmon et al. (2024)			457	United States	RCT	Other (High School)	Digital	None	Individual-based	Prevention
Miller at al. (2021)	14	19	91	United States	RCT	Hospital Clinic	In-person	Yes	Individual-based	Prevention

### Summary of Intervention Characteristics

The studies analyzed varied significantly in their design, session duration, and the components they included (Table 1). Of these, 8 were randomized controlled trials (RCTs) or quasi-randomized trials, while 13 were nonrandomized or open trials with no control condition. The duration of the SSIs varied significantly, ranging from 9 to 300 min, with an average of 75 min across all 21 studies. Among the included studies 10 measured outcomes only immediately following the intervention, 2 at one-month post-intervention, 8 at three months, 4 at six months, and 4 assessed outcomes beyond six months. Most SSIs examined were delivered by providers (*n* = 16), while a smaller number were self-administered by AYAs and did not involve providers (*n* = 5). The SSI studies were primarily carried out in community-based clinics/organizations (*n* = 8). Other SSI studies took place in educational settings, including universities (*n* = 5) and schools (*n* = 6), as well as more specialized locations such as boarding schools (*n* = 1), and police training schools (*n* = 1). The intervention providers were not consistently reported; however, descriptions of providers indicated a range of educational backgrounds, including behavioral health counselors with bachelor’s degrees, registered nurses, and social workers. Most SSIs (*n* = 15) were delivered in group settings, while others (*n* = 6) were delivered individually. Among the group-based SSIs, 14 were presented as educational lectures within school-based courses (rather than as formal ‘treatments’ or ‘interventions’), and 7 were workshop-style sessions conducted in general medical clinic settings. The interventions were implemented through various formats: in-person (*n* = 12), digital (*n* = 7), and hybrid (*n* = 2).

Most of the included studies (*n* = 13) were grounded in specific health and behavioral change theories. These included the Information-Motivation-Behavioral Skills (IMB) Model [56, 57], Social Cognitive Theory (SCT) [58], AIDS Risk Reduction Model (ARRM) [59], Theory of Planned Behavior (TPB) [60], and the Trans-Theoretical Model (TTM) [61]. These studies examined how the change mechanisms proposed within these theories support sustained changes in beliefs, emotions, and behaviors specific to HIV prevention or treatment. A subset of our reviewed studies incorporated mental health-focused components, such as motivational interviewing (*n* = 3), or were adapted from mental health interventions like CBT. Of the 21 SSIs reviewed, two were adaptations of existing multi-session interventions or programs originally designed for specific populations but modified for broader use in different settings or with diverse groups. Three SSIs were web-based, three were entirely video-based (including one played on a loop in a waiting room), and an additional four incorporated video components. Some SSIs featured games or interactive activities, while others were more focused on lessons or education, often involving discussions or printed materials. Several also employed motivational interviewing or counseling approaches.

### HIV-Related Outcomes Represented across Included Studies

Our review found minimal variation among studies in terms of prevention or treatment related outcomes included. Nearly all studies (*n* = 19) focused on prevention, with only two examining treatment-related outcomes [50, 54]. Among the prevention-focused studies, the majority evaluated whether SSIs led to increased HIV testing and improving education about transmission, with one trial testing whether an SSI promoting abstinence lowered HIV risk [37]. Despite the large body of evidence for PrEP as an effective HIV prevention strategy, only one of the 19 prevention studies evaluated outcomes related to PrEP usage [47]. This study found no significant differences between the SSI and control groups in the number of PrEP care visits or initiation rates.

### Effectiveness of SSIs for HIV Treatment Outcomes

The two treatment-focused studies examined the effects of an SSI on different aspects of HIV care. One examined whether an SSI increased treatment initiation and adherence [50], and the second whether an SSI improved adaptive attitudes towards treatment adherence [54]. Notably, these were the only studies among the total reviewed to include transgender and gender-diverse individuals. Both studies demonstrated improved treatment outcomes among participants in the SSI groups compared to the control groups. For instance, the first study was a quasi-experimental trial involving over 2,000 patients across two HIV clinics. Participants in the intervention group watched a 29-minute, theory-based video that depicted individuals overcoming barriers to initiating treatment, adhering to medication regimens, and attending medical appointments. Key outcomes, including HIV viral load test results, were compared between those who viewed the video and those who did not. Results showed a 10.4%-point increase in treatment initiation among patients exposed to the video (60.3–70.7%, *p* < 0.01). Additionally, there was a 6.0%-point improvement in viral suppression rates (56.7–62.7%, *p* < 0.01). However, there was no significant change in return visit rates (77.5–78.8%) [50]. The second study was a small pilot trial involving 18 patients living with HIV at a university-based clinic [58]. Participants engaged in a 30–40-minute motivational interview designed to enhance treatment adherence. Preliminary results showed that four participants (36%) experienced decreases in their adherence attitude scores from pretest to posttest, while two participants (11%) showed no change, and 12 participants (67%) demonstrated improved attitudes toward adherence to antiretroviral therapy. Although the median change in adherence scores suggested a positive trend, the results were not statistically significant (*p* = 0.08).

### Effectiveness of SSIs for HIV Prevention Outcomes

As summarized in Table 2, the most measured outcome across included SSI studies was HIV education (i.e., knowledge of HIV) (present in *n* = 13 included studies). This outcome was typically assessed using a composite score derived from existing or adapted tools to evaluate participants’ knowledge on key topics, such as HIV transmission and testing methods, at post-test. Among the 13 studies that assessed HIV knowledge, all reported high scores for participants who received an SSI (*n* = 5 nonrandomized studies; *n* = 8 RCTs). While most studies followed a pre/post-test design, five included additional follow-up assessments spanning 3 to 12 months. These follow-ups revealed that SSI recipients consistently outperformed the control group on HIV education measures at various intervals, including 3 months, 6 months, and 1-year post-SSI.

**Table 2:** Reviewed articles (continued)

Article	Theory Mentioned	Length of intervention (minutes)	Follow-Up Periods	Comparator(s)/Control	HIV Primary Outcomes	Psychosocial Outcome Mentioned	Outcome(s) Specific to Adolescent Age Range
Abdullah et al. (2005)	Information-Motivation-Behavioral Skills Model (IMB)	90 min	Baseline, Post Intervention, 4 m	No-Treatment	HIV Knowledge HIV Attitudes HIV Perceived Risk Sexual Health Self Efficacy	NA	Participants in the experimental group (SSI recipients) showed increased HIV knowledge, more positive attitudes toward HIV prevention, improved risk appraisal, and greater likelihood of discussing safer sexual health practices, including HIV testing, with partners and friends
Abolfotouh (1995)	N/A	Unspecified	Baseline	No-Treatment	HIV Knowledge HIV Perceived Risk	NA	65% of the experimental group (SSI recipients) showed increased HIV knowledge. Both groups were less aware that HIV cannot spread through casual contact, but the experimental group reported significantly less fear of contracting HIV compared to the control group
Agha and Van Rossem (2004)	N/A	105 min	Baseline, 2 m, 8 m	Other Non-Active Comparator	HIV Attitudes	NA	Participants in the experimental group showed higher awareness than the control group, believing that HIV can be avoided through abstinence, that healthy-looking individuals can have HIV, and that HIV is incurable
Ashworth et al. (1992)	N/A	60 min	Baseline, 2 Weeks	No- Treatment	HIV Knowledge HIV Perceived Risk	NA	Participants who received the SSI showed significantly higher knowledge at posttest, were less worried about HIV exposure, but expressed increased concern about HIV acquisition in adulthood, even after accounting for prior HIV education
Ballester-Arnal et al. (2017)	Unspecified Behavioral Change Theories (N/A)	180 min	Baseline, 1 Week, 1 M, 3 M, and 1 Year	No Control	HIV Knowledge HIV Attitudes HIV Perceived Risk	NA	Participants in the experimental group showed increased HIV education post-intervention (sustained over time), heightened fear of HIV infection at follow-ups, a shift in perception of HIV/AIDS from fatal to high severity, and progressively higher HIV susceptibility perception from pre-intervention (17.66%) to short-term (20.93%), medium-term (20.91%), and long-term (22.21%)
Bangi et al. (2013)	AIDS Risk Reduction Model	300 min	Baseline, 3 m	Other Non-Active Comparator	HIV Knowledge HIV Attitudes HIV Perceived Risk		Participants who completed the SSI demonstrated greater knowledge of HIV/STI prevention and protection, enhanced understanding of living with HIV, and an increased awareness of their perceived risk of HIV
Brus and Jennit (2009)	Social Cognitive Theory	60–120 min	Baseline, Post Intervention	No Control	HIV Knowledge HIV Attitudes	NA	Before the session, participants held several misconceptions about HIV. After completing the SSI, correct survey scores improved by 24%. Stigmatizing attitudes toward HIV decreased significantly, dropping from 38–29%. Additionally, the percentage of students who recognized the importance of HIV education increased from 80–90%
Ergene et al. (2005)	N/A	75 min	Baseline, Post Intervention	W aitlist	HIV Knowledge HIV Attitudes	NA	Participants who received the standard SSI or an enhanced version demonstrated greater success in improving students’ knowledge about HIV and attitudes toward individuals living with HIV compared to those in the control group
Harper et al. (2009)	AIDS Risk Reduction Model (ARRM)	120 min	Post-intervention and 2 month	Other Non-Active Comparator	HIV/AIDS knowledge Sexual Health Self Efficacy	Self-esteem	Participants who received the SSI demonstrated improved knowledge about HIV compared to the control group. The control group reported a higher ability to discuss HIV-related health concerns with a sexual partner than those in the experimental group (who received the SSI). As for self-esteem, participants in the SSI group showed gradual improvement over time
Jenner et al. (2023)	Elaboration Likelihood Model (E-ELM) & Social Cognitive Theory (SCT)	23 min	3 months	Other Non-Active Comparator	Intentions to Test for HIV HIV Perceived Risk	NA	Three months after completing the SSI, participants in the experimental group were more likely to undergo HIV testing and demonstrated a heightened, more accurate awareness of their HIV risk
Kamke et al. (2020)	Reasoned Action Model	Average of 44 min	Pre-/post-test	Psychoeducational	HIV Education Sexual Health Self Efficacy	NA	At posttest, participants who received the SSI demonstrated significantly greater intentions to communicate, improved communication skills for HIV prevention, enhanced STI/HIV knowledge, and higher sexual self-efficacy compared to the comparison group
Karnik et al. (2023)	Health Belief Model	Average of under 10 min	1, 3, 6, and 12-month time points	Psychoeducational	At least 1 PrEP care visit in a 3-month period	Substance Use	There were no notable differences in the number of PrEP care visits between the groups. Additionally, the intervention did not have a statistically significant impact on substance use or any other measured outcomes across all time points
Kennedy et al. (2013)	Social Cognitive Theory (SCT) Trans-Theoretical Model (TTM)	45–60 min	Baseline, 3 month, 6 month	Other Non-Active Comparator	HIV Perceived Risk	NA	At each time point, participants who received the SSI exhibited higher scores in perceived HIV susceptibility
Neumann et al. (2018)	Social Cognitive Theory, Information-Motivation-Behavioral Skills model, and Social Action Theory	29 min	(Before/within 7 days after index visit), After 3 months of follow-up from index visit, at 183 days or 6 months	Other (Historical Condition)	ART Medication adherence. HIV Treatment Initiation	NA	The initiation of HIV treatment showed a notable overall increase of 10.4% points between the study periods, alongside improved rates of viral suppression among individuals who engaged with the SSI
O’Grady et al. (2009)	Information Motivation Model	1 h	Pre-test & post-test	Other Non-Active Comparator	Intentions to Test for HIV HIV Knowledge Sexual Health Self Efficacy	NA	The results indicated that participants who received the SSI demonstrated greater preventative knowledge compared to the control group. Furthermore, the analysis revealed that the intervention effectively boosted motivation for engaging in safer sexual behaviors. However, no significant impact was observed on participants’ perceived ability to perform preventative behaviors as a result of the intervention
Roye et al. (2007)	Social Cognitive Theory, Theory of Reasoned Action, and the Health Belief Model	40–45 min	3 months, 12 months	Usual Care	HIV Perceived Risk	NA	No significant differences were observed between the groups in how participants perceived their risk of HIV
Widman et al. (2020)	Reasoned Action Model	45 min	Pre/post-test	Other Non-Active Comparator	HIV Knowledge Sexual Health Self Efficacy	NA	After completing the SSI, students showed significant improvements in sexual communication intentions, HIV/STD knowledge, self-efficacy in practicing safer sex, and sexual assertiveness compared to those in the control group
Yeagley et al. (2012)	Trans Theoretical Model	36–90 min	During same clinic visit	No Control	Attitudes regarding adherence to ART medication	NA	Overall, four participants (36%) showed a decline in their attitudes toward adherence from pre- to posttest, indicating a worsening perspective on adherence. Two participants (11%) maintained the same scores, while 12 (67%) demonstrated improved attitudes toward adhering to antiretroviral medications following the intervention. However, the median change in adherence scores between pre- and posttest was not statistically significant (*p* = 0.08). Regarding attitudes toward disclosure, four participants (22%) exhibited a decline in their scores, reflecting more negative views on disclosure. Three participants (17%) showed no change, while 11 (61%) reported improved attitudes toward disclosure after the SSI intervention
Jemmott et al. (1992)	N/A	300	3-month follow up	Other Non-Active Comparator	HIV Knowledge	NA	Following the intervention, participants who completed the SSI demonstrated a significant increase in their knowledge of HIV/AIDS. Even three months later, their scores on HIV/AIDS knowledge remained higher than those of participants in the control group
McCrimmon et al. (2024)	Reasoned Action Model (RAM; Fishbein & Ajzen, 2010)	45	Pre- and post- test only	Psychoeducational	HIV Knowledge Sexual Health Self Efficacy	NA	After the intervention, participants who completed the SSI demonstrated notable improvements in sexual assertiveness, intentions to communicate about sexual matters, HIV/STI knowledge, and confidence in practicing safer sex
Miller at al. (2021)	Theory of Planned Behavior, Social Ecological Model	24.6 min	Baseline, post-intervention, 6 month follow-up	Other Non-Active Comparator	Intentions to Test for HIV	NA	Compared with controls, participants who received the SSI were more likely to complete 1 service including HIV testing. There were no meaningful differences between arms in behaviors at follow-up

*Notes for studies added outside systematic review*: Jemmott et al. 1992) Added via review of past lab meta-analysis/umbrella review studies; specifically, our umbrella review (https://osf.io/preprints/psyarxiv/gp6sx) referenced Kalichman et al. 1996 (10.1007/BF02903934) which in turn reviewed Jemmott et al. 1992, McCrimmon et al. /92024) Newly-released study, Miller et al. (2021) Dissertation excluded from review; this newer work built upon it

The second most assessed outcome across the studies was perceived HIV risk or perceived susceptibility to HIV (*n* = 10). Measurement methods varied, some studies utilized a single-item approach (e.g., “What is the likelihood or risk you perceive of getting HIV?” or “How worried or concerned are you about getting HIV/AIDS from having sex?“), while others calculated a summed score estimating probabilities of HIV infection from both everyday activities and high-risk behaviors. Of the ten studies assessing SSI impacts on perceived susceptibility, eight reported that completing an SSI was associated with (*n* = 2 nonrandomized studies) or led to (in 6 = RCTs) more accurate risk perception at post-intervention. Additionally, as shown in Table 2, five of these studies demonstrated sustained improvements in risk perception during follow-up evaluations at various intervals.

The third most frequently assessed outcomes in the studies were sexual health self-efficacy and HIV attitudes. For sexual health self-efficacy, researchers measured participants’ perceived ability and confidence to discuss HIV prevention, or their intentions to talk about HIV and safer sexual practices with main or casual sex partners. These assessments were conducted using either a single-item measure or composite scores based on multiple questions. Eight studies reported improvements in participants’ confidence and intentions to discuss HIV and sexual health with their partners immediately following the intervention (*n* = 2 nonrandomized studies; *n* = 6 RCTs). Studies examining HIV-related attitudes used various measures but generally focused on three key areas: (1) perceptions of HIV as either a curable or fatal condition, (2) attitudes toward HIV prevention, and (3) attitudes toward individuals living with HIV. Seven studies assessed these attitudes reported improvements, with three demonstrating sustained, statistically significant positive changes among participants in the intervention group compared to the control group (*n* = 1 nonrandomized study; *n* = 0 RCTs). These results highlight a meaningful shift in perceptions, as participants began to see HIV less as a fatal illness and more as a manageable condition. Furthermore, their attitudes toward individuals living with HIV became notably less stigmatizing.

HIV testing, both actual testing and the intention to test, was assessed in three of the 21 studies as a single metric (e.g., “After completing the activity today, how likely are you to get an HIV test within the next 3 months?”). All three studies reported that completing an SSI either resulted in (*n* = 2, RCTs) or was associated with (*n* = 1, nonrandomized study) increased intentions to get tested for HIV AYAs compared to those in a control group. One of these studies included a three-month follow-up, where participants were assigned to either a single-session entertainment-education sexual health video intervention or a control video [49]. Participants who viewed the sexual health video demonstrated borderline elevated perceptions of HIV/STI risk (*p* = 0.057) and a greater likelihood of getting HIV tested (*p* = 0.073) at three months.

### Mental Health Outcomes

To evaluate the extent to which these interventions address mental health outcomes, each study was reviewed and coded based on two criteria: (1) whether the SSI included any reference to mental health, and (2) whether the study assessed any mental health-related outcomes. All 21 studies referenced a mental health component, such as addressing emotion regulation challenges or substance use issues among AYA or incorporating mental health screeners as part of their inclusion criteria. However, only two of these studies went beyond mentioning mental health to include specific mental health-related outcomes. The first study examined self-esteem using a 10-item scale, comparing participants who completed a multi-session HIV prevention program, primarily focused on improving HIV education and sexual health self-efficacy, with those who attended a single, two-hour information-based session [47]. Results indicated that both groups reported improvements in self-esteem post-intervention, suggesting a positive effect regardless of program intensity. The second study was an efficacy trial evaluating an SSI designed to address alcohol misuse among AYAs undergoing HIV testing in comparison to a time- and attention-matched control condition [51]. The SSI utilized motivational content delivered via a digital platform, including topics such as the importance of change, the downsides of drinking, and barriers to change. The content was presented through interactive slides with elements such as checkboxes, dropdown menus, and short text fields for participants to engage in brief exercises. While the SSI did not produce statistically significant reductions in problematic drinking across all time points, it showed promise in reducing HIV risk behaviors. Specifically, participants in the intervention group reported a decrease in condomless insertive anal sex under the influence at the 12-month follow-up compared to the control group.

## Discussion

We conducted the first comprehensive review synthesizing existing research on SSIs targeting HIV-related outcomes among AYAs. Considering the close relationship between the HIV epidemic and mental health challenges, we also examined the extent to which these SSIs addressed mental health-related outcomes. While prior reviews have highlighted the potential of SSIs to reduce sexual risk behaviors, such as unprotected sex and inconsistent condom use across diverse populations and settings, our review specifically examines HIV-related outcomes, including HIV education, PrEP initiation, HIV testing, and treatment initiation and adherence. Of the 21 studies included, 19 evaluated SSIs focused on HIV prevention efforts. These studies reported small to moderate but statistically significant effects across multiple HIV prevention measures. Specifically, findings from randomized and nonrandomized trials indicated that SSIs increased knowledge of HIV prevention and transmission, reduced stigmatizing attitudes toward individuals living with HIV, and enhanced self-assessment of HIV risk. Although only two studies directly addressed HIV treatment, the results were promising, showing positive outcomes in facilitating treatment initiation and improving attitudes related to treatment adherence. Notably, only two of the 21 studies included mental health outcomes, despite all SSIs being grounded in mental health interventions and behavioral change theories.

The findings have significant implications for clinical practice and public health in the context of HIV care. SSIs offer a cost-effective and impactful strategy to extend the reach of HIV treatment and prevention efforts, particularly for individuals who are unable or hesitant to engage with more intensive support programs. When compared to multi-session HIV prevention and treatment initiatives, SSIs demonstrate comparable effectiveness in certain cases, especially in improving prevention-related outcomes, as summarized in this review. As has been emphasized in reviews suggesting SSIs’ effectiveness for mental health problems, current findings do *not* suggest that multi-session HIV programs should be replaced by SSIs [62]. Instead, addressing the diverse needs of populations affected by HIV will require a multi-tiered approach that integrates various forms of support. The findings underscore the versatility and potential of SSIs to benefit diverse populations and settings, as demonstrated by the wide range of SSIs reviewed. However, the results also highlight areas needing further exploration, particularly the integration of mental health components into HIV-focused SSIs. Future research should aim to address these gaps to optimize the effectiveness and reach of SSIs for populations affected by HIV.

Regarding the delivery formats of SSIs reviewed, the majority (16 out of 21) were designed to be implemented by a trained provider, such as behavioral health counselors, nurses, or peer educators trained to deliver the intervention. These interventions were delivered in both individual (*n* = 6) and group format (*n* = 15). Of these, 12 were designed for face-to-face delivery by a provider, seven were entirely digital, and two were hybrid interventions, incorporating both self-guided components and some level of in-person interaction. While provider-delivered SSIs have demonstrated effectiveness across various HIV-related outcomes, scaling these interventions poses significant challenges [63]. Provider-led SSIs require trained personnel for implementation, which can increase the burden on clinics. In contrast, self-guided SSIs offer a more scalable and resource-efficient alternative, as they do not require formal or lay providers. For example, one entirely fully self-administered SSI included in this review is *HEART* (*Health Education and Relationship Training)*, a brief, digital sexual health intervention designed to be completed in approximately 45 min [49]. The program includes five modules aligned with the Reasoned Action Model’s [64] theory-based domains of sexual health behavior: (a) safer sex motivation, (b) STD/HIV knowledge, (c) sexual attitudes and norms, (d) safer sex self-efficacy, and (e) sexual communication skills. Participants in the Results indicated that *HEART* was effective in increasing sexual assertiveness, sexual communication intentions, HIV/STI knowledge, condom attitudes, and safer sex self-efficacy when compared to the control condition. While no SSIs included in our review directly compared HIV-focused SSIs to traditional multisession interventions, evidence from a meta-analysis of menta health focused SSIs suggests comparable outcomes between self-guided and provider-led approaches in reducing symptom severity (mean *g* = 0.32 across both modalities) [31]. This finding points to the potential for HIV-focused SSIs to be equally effective across certain outcomes, particularly given that our review captured similar positive effects observed in multisession HIV interventions across various time points [65]. However, further research is needed to directly compare HIV-focused SSIs to traditional multisession programs to confirm these potential impacts.

The reviewed SSIs were primarily psychoeducational, typically delivered through lectures or videos. These formats offered limited opportunities for participants to actively engage in identifying, applying, or practicing key skills often emphasized in more comprehensive, evidence-based HIV programs. Such skills include recognizing barriers to HIV testing and developing actionable testing plans. While psychoeducational approaches are effective in increasing knowledge, they tend to have less impact on driving motivation or fostering long-term behavior change [66]. Research on SSIs aimed at improving mental health outcomes suggests that interventions incorporating skill-building components, rather than relying solely on psychoeducation or resource distribution, achieve stronger results in both the short and long term [25].Skill-building activities can enhance critical factors such as hope, self-efficacy, and a sense of control—key mechanisms of change identified in mental health-focused SSIs [24]. To improve existing HIV-focused SSIs, incorporating interactive elements that effectively influence HIV/AIDS-related behaviors over short, medium, and long-term periods may be useful. For example, one study from our review demonstrated success by using interactive exercises (e.g., completing unfinished sentences), real testimonials from individuals living with HIV, and self-guided activities designed to build skills like refusing risky sexual behavior. This approach led to significant improvements in HIV knowledge, perception of susceptibility, and sexual health self-efficacy from pre-test to follow-up evaluations. Although few SSIs in this review included such opportunities, expanding their format to include interactive elements may enhance their overall effectiveness in addressing both HIV and mental health outcomes. Future research should further explore integrating such interactive components into HIV-focused SSIs to maximize their impact on behavior change over time.

The effectiveness of SSIs on HIV treatment-related outcomes remains difficult to generalize, as only two studies in our review specifically addressed key outcomes such as initiating treatment, improving medication adherence, or fostering positive attitudes toward adherence. This observation aligns with recent reviews that highlight the limited availability of brief interventions targeting HIV treatment outcomes [67]. Additionally, only two studies in our review incorporated a mental health outcome, none of which were directly tied to treatment-focused interventions. This highlights a critical gap: the need for SSIs that integrate mental health support with HIV treatment outcomes. Such integration is particularly important given the significant impact mental health challenges can have on treatment success [22]. For example, a multisite study in the United States found that mental health issues, such as depression, are strongly linked to lower adherence to ART [11]. Depressed patients exhibited a 42% lower adherence rate to ART, yet few of these patients report receiving formal services for their depression symptoms underscoring the urgent need for scalable, accessible supports that can address both mental health and HIV outcomes simultaneously [11]. Given this gap, developing SSIs that address both HIV treatment and mental health outcomes presents a promising opportunity. While the studies reviewed did not target both outcomes simultaneously, they frequently used similar theories of change and behavior change techniques. For instance, many SSIs for HIV and mental health are grounded in shared theoretical frameworks, such as the transtheoretical model or theory of planned behavior, which emphasize mechanisms like self-monitoring, behavioral activation, and reinforcement [68]. A dual-purpose SSI could capitalize on these shared mechanisms. Such an intervention could, for example, integrate self-monitoring for both medication management and behavioral activation, using reflection, cues to action, and reinforcement to enhance self-awareness, build motivation, and promote skill development [69]. These strategies could address both ART adherence and mental health outcomes, such as reducing depression, through the same underlying mechanisms of behavior change. Future research should prioritize the development and evaluation of integrated SSIs that address both HIV and mental health outcomes.

In summary, this review represents the first overview of the clinical utility of SSIs for AYA in improving specific HIV-specific outcomes. Previous reviews have primarily focused on the effectiveness of SSIs in reducing sexual risk behaviors among AYA, leaving a gap in understanding their broader impact. While we utilized a rigorous approach to identify relevant literature, it is possible that some studies employing a “single session” model were missed. Notably, three of the 21 studies included in this review were identified outside the systematic search process. Furthermore, there is limited evidence on the effectiveness of integrated SSIs combining HIV treatment and mental health components for AYA. Despite these limitations, our findings underscore the potential of SSIs to achieve targeted outcomes, such as improving knowledge about HIV transmission and prevention, fostering positive attitudes towards people living with HIV, and enhancing sexual self-efficacy. These brief interventions demonstrate promise in creating meaningful improvements in HIV-related treatment and prevention outcomes. However, to maximize their impact and effectively reach high-risk populations, further steps are needed. One potential solution is adapting proven SSIs to include interactive elements or design features that go beyond psychoeducation, for example, incorporating opportunities to practice skills or testimonials from peers [24]. Additionally, only one of the seven studies published since the introduction of PrEP in 2012 included PrEP-related outcomes. This underscores the need to expand SSIs to integrate recent biomedical advances in HIV care. Ultimately, SSIs offer a concise, resource-efficient format that can benefit researchers, community organizations, and participants alike. By incorporating the recommendations from this review, such as integrating interactive components and addressing contemporary biomedical approaches, SSIs can be further refined and adapted to achieve greater effectiveness, while avoiding the challenges of participant retention often associated with multi-session interventions.

## Supplementary Information

Below is the link to the electronic supplementary material.

Supplementary Material 1

## Data Availability

Not Applicable.
