# Supplementary material for: Single-Session Interventions To Enhance HIV Outcomes among Adolescents and Young Adults: A Systematic Scoping Review and Implications for Integrating HIV and Mental Health Services
Source: AIDS Behav. 2025 Aug 1;29(12):3981–98. doi: 10.1007/s10461-025-04834-4 (PMC12580434; doi:10.1007/s10461-025-04834-4)
Supplement: Supplementary file 1 — Supplementary Material 1 [file 10461_2025_4834_MOESM1_ESM.docx]

Supplementary File 1. Preferred Reporting Items for Systematic reviews and Meta-Analyses extension for Scoping Reviews (PRISMA-ScR) Checklist

| **SECTION** | **ITEM** | **PRISMA-ScR CHECKLIST ITEM** | **REPORTED**  **ON PAGE #** |
| --- | --- | --- | --- |
| **TITLE** | | | |
| Title | 1 | Identify the report as a scoping review. | 1 |
| **ABSTRACT** | | | |
| Structured summary | 2 | Provide a structured summary that includes (as applicable): background, objectives, eligibility criteria, sources of evidence, charting methods, results, and  conclusions that relate to the review questions and objectives. | 2 |
| **INTRODUCTION** | | | |
| Rationale | 3 | Describe the rationale for the review in the context of what is already known. Explain why the review  questions/objectives lend themselves to a scoping review approach. | 3-4 |
| Objectives | 4 | Provide an explicit statement of the questions and objectives being addressed with reference to their key elements (e.g., population or participants, concepts, and context) or other relevant key elements used to  conceptualize the review questions and/or objectives. | 4 |
| **METHODS** | | | |
| Protocol and registration | 5 | Indicate whether a review protocol exists; state if and where it can be accessed (e.g., a Web address); and if available, provide registration information, including the registration number. | 4 |
| Eligibility criteria | 6 | Specify characteristics of the sources of evidence used as eligibility criteria (e.g., years considered, language,  and publication status), and provide a rationale. | 5 |
| Information sources* | 7 | Describe all information sources in the search (e.g., databases with dates of coverage and contact with authors to identify additional sources), as well as the date the most recent search was executed. | 5 |
| Search | 8 | Present the full electronic search strategy for at least 1  database, including any limits used, such that it could be repeated. | Supplementary File 2 |
| Selection of sources of evidence† | 9 | State the process for selecting sources of evidence (i.e., screening and eligibility) included in the scoping review. | 5-6 |
| Data charting process‡ | 10 | Describe the methods of charting data from the included sources of evidence (e.g., calibrated forms or forms that have been tested by the team before their use, and whether data charting was done independently or in duplicate) and any processes for obtaining and  confirming data from investigators. | 5 |
| Data items | 11 | List and define all variables for which data were sought and any assumptions and simplifications made. | 5 |
| Critical appraisal of individual sources of evidence§ | 12 | If done, provide a rationale for conducting a critical appraisal of included sources of evidence; describe the  methods used and how this information was used in any data synthesis (if appropriate). | N/A |
| Synthesis of results | 13 | Describe the methods of handling and summarizing the data that were charted. | 5 |

**Supplementary File 2. Search Terms**

| **Database searched** | **Date searched** | **Results** |
| --- | --- | --- |
| Ovid MEDLINE | 26 June 2024 | **428** |
| Cochrane Database of Systematic Reviews (Wiley) | 26 June 2024 | **21** |
| Cochrane Central Register of Controlled Trials (Wiley) | 26 June 2024 | **510** |
| APA PsycINFO (Ebsco) | 26 June 2024 | **148** |
| Web of Science (Clarivate) | 26 June 2024 | **429** |
| ProQuest Dissertations & Theses Global | 26 June 2024 | **56** |
| Total | | **1592** |
| After de-duplication | | **1116** |

**Example Search Methods:**

The review authors partnered with a research librarian (ABW) to create a comprehensive search of the literature. The following databases were searched on 26 June 2024: Ovid MEDLINE, Cochrane Library (Wiley), APA PsycINFO (Ebsco), Web of Science (Clarivate), and ProQuest Dissertation and Theses Global. The search strategy included a combination of controlled vocabulary and keyword searching related to brief interventions for adolescents with HIV. All databases were searched from inception to present without the use of filters or limits. Records were downloaded and underwent multi-pass deduplication in a citation management software (EndNote) and unique records were uploaded to a screening platform (Rayyan) for initial screening by two independent reviewers.

| **Ovid MEDLINE(R) ALL <1946 to June 25, 2024>** | | |
| --- | --- | --- |
| **Number** | **Search** | **Results** |
| 1 | ((Single or brief) adj5 (session* or therap* or intervention* or appointment* or program* or workshop*)).ti,ab. | 61524 |
| 2 | ((One or standalone or stand-alone) adj (session or appointment or therap* or intervention)).ti,ab. | 9211 |
| 3 | Hour-session.ti,ab. | 507 |
| 4 | 1 or 2 or 3 | 70445 |
| 5 | exp HIV/ | 109334 |
| 6 | exp HIV Infections/ | 323448 |
| 7 | (Human-immunodeficienc*-vir* or acquired-immunodeficienc*-syndrome* or HIV or HIV-1* or HIV1* or HIV-2* or HIV2* or AIDS or PLWH or YLWH).ti,ab. | 473249 |
| 8 | ((ART or HAART or anti-retroviral-therap* or antiretroviral-therap*) adj2 adherence).ti,ab. | 4450 |
| 9 | (Condomless* or condom-use or unprotected-sex* or sexual-risk*).ti,ab. | 21800 |
| 10 | 5 or 6 or 7 or 9 | 518530 |
| 11 | exp Adolescent/ | 2255991 |
| 12 | exp Adolescent Behavior/ | 35864 |
| 13 | exp Psychology, Adolescent/ | 13843 |
| 14 | (Adolescen* or older-child* or Teen or Teens or Teenager* or Youth* or Elementary-school* or Middle-school* or High-school* or School-age* or Preteen or Preteens or Preteenager* or Prepub* or Puberty or Pubescen* or Preadolescen* or juvenile*).ti,ab. | 653461 |
| 15 | (Young adj2 (person* or people* or patient*)).ti,ab. | 95574 |
| 16 | 11 or 12 or 13 or 14 or 15 | 2578869 |
| 17 | 4 and 10 and 16 | 428 |

| **Cochrane Central Register of Controlled Trials & Cochrane Database of Systematic Reviews** | | |
| --- | --- | --- |
| **Number** | **Search** | **Results** |
| #1 | (((Single OR brief) NEAR/5 (session* OR therap* OR intervention* OR appointment* OR program* OR workshop*))):ti,ab,kw | 32874 |
| #2 | (((One OR standalone OR stand-alone) NEAR (session OR appointment OR therap* OR intervention))):ti,ab,kw | 45537 |
| #3 | ((Hour-session)):ti,ab,kw | 530 |
| #4 | #1 OR #2 OR #3 | 75675 |
| #5 | MeSH descriptor: [HIV] explode all trees | 4231 |
| #6 | MeSH descriptor: [HIV Infections] explode all trees | 17783 |
| #7 | ((Human-immunodeficiency-vir* OR Human-immunodeficiencies-vir* OR acquired-immunodeficiency-syndrome* OR acquired-immunodeficiencies-syndrome* OR HIV OR HIV-1 OR HIV1 OR HIV-2 OR HIV2 OR AIDS OR PLWH OR YLWH)):ti,ab,kw | 39179 |
| #8 | (((ART OR HAART OR anti-retroviral-therap* OR antiretroviral-therap*) adj2 adherence)):ti,ab,kw | 15367 |
| #9 | ((Condomless* OR condom-use OR unprotected-sex* OR sexual-risk*)):ti,ab,kw | 3150 |
| #10 | #5 OR #6 OR #7 OR #8 OR #9 | 45671 |
| #11 | MeSH descriptor: [Adolescent] explode all trees | 137144 |
| #12 | MeSH descriptor: [Adolescent Behavior] explode all trees | 1960 |
| #13 | MeSH descriptor: [Psychology, Adolescent] explode all trees | 313 |
| #14 | ((Adolescen* OR older-child* OR Teen OR Teens OR Teenager* OR Youth* OR Elementary-school* OR Middle-school* OR High-school* OR School-age* OR Preteen OR Preteens OR Preteenager* OR Prepub* OR Puberty OR Pubescen* OR Preadolescen* OR juvenile*)):ti,ab,kw | 188139 |
| #15 | ((Young NEAR/2 (person* or people* or patient*))):ti,ab,kw | 6997 |
| #16 | #11 OR #12 OR #13 OR #14 OR #15 | 191743 |
| #17 | #4 AND #10 AND #16 | **531** |
|  |  |  |

| **APA Psycinfo (Ebsco)** | | |
| --- | --- | --- |
| **Number** | **Search** | **Result** |
| S1 | TI ( ((Single or brief) N5 (session* or therap* or intervention* or appointment* or program* or workshop*)) ) OR AB ( ((Single or brief) N5 (session* or therap* or intervention* or appointment* or program* or workshop*)) ) | 28,199 |
|  |  |  |
|  |  |  |
| S2 | TI ( ((One or standalone or stand-alone) N0 (session or appointment or therap* or intervention)) ) OR AB ( ((One or standalone or stand-alone) N0 (session or appointment or therap* or intervention)) ) | 9,216 |
|  |  |  |
|  |  |  |
| S3 | TI Hour-session OR AB Hour-session | 649 |
|  |  |  |
|  |  |  |
| S4 | S1 OR S2 OR S3 | 37,242 |
|  |  |  |
|  |  |  |
| S5 | DE "HIV" | 42,044 |
|  |  |  |
|  |  |  |
| S6 | DE "AIDS" | 15,966 |
|  |  |  |
|  |  |  |
| S7 | TI ( (Human-immunodeficienc*-vir* or acquired-immunodeficienc*-syndrome* or HIV or HIV-1* or HIV1* or HIV-2* or HIV2* or AIDS or PLWH or YLWH) ) OR AB ( (Human-immunodeficienc*-vir* or acquired-immunodeficienc*-syndrome* or HIV or HIV-1* or HIV1* or HIV-2* or HIV2* or AIDS or PLWH or YLWH) ) | 78,227 |
|  |  |  |
|  |  |  |
| S8 | TI ( ((ART or HAART or anti-retroviral-therap* or antiretroviral-therap*) N2 adherence) ) OR AB ( ((ART or HAART or anti-retroviral-therap* or antiretroviral-therap*) N2 adherence) ) | 1,920 |
|  |  |  |
|  |  |  |
| S9 | TI ( Condomless* or condom-use or unprotected-sex* or sexual-risk* ) OR AB ( Condomless* or condom-use or unprotected-sex* or sexual-risk* ) | 14,321 |
|  |  |  |
|  |  |  |
| S10 | S5 OR S6 OR S7 OR S8 OR S9 | 83,882 |
|  |  |  |
|  |  |  |
| S11 | DE "Adolescent Behavior" | 20,731 |
|  |  |  |
|  |  |  |
| S12 | DE "Adolescent Psychology" | 6,071 |
|  |  |  |
|  |  |  |
| S13 | TI ( Adolescen* OR older-child* OR Teen OR Teens OR Teenager* OR Youth* OR Elementary-school* OR Middle-school* OR High-school* OR School-age* OR Preteen OR Preteens OR Preteenager* OR Prepub* OR Puberty OR Pubescen* OR Preadolescen* OR juvenile* ) OR AB ( Adolescen* OR Teen OR Teens OR Teenager* OR Youth* OR Elementary-school* OR Middle-school* OR High-school* OR School-age* OR Preteen OR Preteens OR Preteenager* OR Prepub* OR Puberty OR Pubescen* OR Preadolescen* OR juvenile* ) | 502,509 |
|  |  |  |
|  |  |  |
| S14 | TI ( (Young N2 (person* or people* or patient*)) ) OR AB ( (Young N2 (person* or people* or patient*)) ) | 47,564 |
|  |  |  |
|  |  |  |
| S15 | S11 OR S12 OR S13 OR S14 | 527,978 |
|  |  |  |
|  |  |  |
| S16 | S4 AND S10 AND S15 | 148 |
|  |  |  |
|  |  |  |

| **Web of Science (Clarivate)** | | |
| --- | --- | --- |
| **Number** | **Search** | **Result** |
| 1 | TS=(((Single or brief) NEAR/5 (session* or therap* or intervention* or appointment* or program* or workshop*))) | 87250 |
| 2 | TS=((One or standalone or stand-alone) NEXT (session or appointment or therap* or intervention)) | 17635 |
| 3 | TS=Hour-session | 2171 |
| 4 | #3 OR #2 OR #1 | 106572 |
| 5 | TS=(Human-immunodeficienc*-vir* or acquired-immunodeficienc*-syndrome* or HIV or HIV-1* or HIV1* or HIV-2* or HIV2* or AIDS or PLWH or YLWH) | 1195861 |
| 6 | TS=((ART or HAART or anti-retroviral-therap* or antiretroviral-therap*) NEAR/2 adherence) | 5152 |
| 7 | TS=(Condomless* or condom-use or unprotected-sex* or sexual-risk*) | 27654 |
| 8 | #5 OR #6 OR #7 | 1203949 |
| 9 | TS=(Adolescen* or Teen or Teens or Teenager* or Youth* or Elementary-school* or Middle-school* or High-school* or School-age* or Preteen or Preteens or Preteenager* or Prepub* or Puberty or Pubescen* or Preadolescen* or juvenile*) | 1259565 |
| 10 | TS=(Young NEAR/2 (person* or people* or patient*)) | 220632 |
| 11 | #9 OR #10 | 1421064 |
| 12 | #4 AND #8 AND #11 | 429 |

| **ProQuest Dissertations & Theses Global** |
| --- |
| Search Strategy |
| Set#: S1 |
| Searched for: title((Single or brief) NEAR/5 (session* or therap* or intervention* or appointment* or program* or workshop*)) OR abstract((Single or brief) NEAR/5 (session* or therap* or intervention* or appointment* or program* or workshop*)) |
| Databases: ProQuest Dissertations & Theses Global |
| Results: 9861 |
|  |
| Set#: S2 |
| Searched for: title((One or standalone or stand-alone) NEXT (session or appointment or therap* or intervention)) OR abstract((One or standalone or stand-alone) NEXT (session or appointment or therap* or intervention)) |
| Databases: ProQuest Dissertations & Theses Global |
| Results: 5017 |
|  |
| Set#: S3 |
| Searched for: title(Hour-session ) OR abstract(Hour-session ) |
| Databases: ProQuest Dissertations & Theses Global |
| Results: 175 |
|  |
| Set#: S4 |
| Searched for: [S1] OR [S2] OR [S3] |
| Databases: ProQuest Dissertations & Theses Global |
| These databases are searched for part of your query. |
| Results: 14934 |
|  |
| Set#: S5 |
| Searched for: title((Human-immunodeficienc*-vir* or acquired-immunodeficienc*-syndrome* or HIV or HIV-1* or HIV1* or HIV-2* or HIV2* or AIDS or PLWH or YLWH) ) OR abstract((Human-immunodeficienc*-vir* or acquired-immunodeficienc*-syndrome* or HIV or HIV-1* or HIV1* or HIV-2* or HIV2* or AIDS or PLWH or YLWH) ) |
| Databases: ProQuest Dissertations & Theses Global |
| Results: 115773 |
|  |
| Set#: S6 |
| Searched for: title((ART or HAART or anti-retroviral-therap* or antiretroviral-therap*) NEAR/2 (adherence) ) OR abstract((ART or HAART or anti-retroviral-therap* or antiretroviral-therap*) NEAR/2 (adherence) ) |
| Databases: ProQuest Dissertations & Theses Global |
| Results: 358 |
|  |
| Set#: S7 |
| Searched for: title((Condomless* or condom-use or unprotected-sex* or sexual-risk*) ) OR abstract((Condomless* or condom-use or unprotected-sex* or sexual-risk*) ) |
| Databases: ProQuest Dissertations & Theses Global |
| Results: 3123 |
|  |
| Set#: S8 |
| Searched for: [S5] OR [S6] OR [S7] |
| Databases: ProQuest Dissertations & Theses Global |
| These databases are searched for part of your query. |
| Results: 116900 |
|  |
| Set#: S10 |
| Searched for: title((Adolescen* or older-child* or Teen or Teens or Teenager* or Youth* or Elementary-school* or Middle-school* or High-school* or School-age* or Preteen or Preteens or Preteenager* or Prepub* or Puberty or Pubescen* or Preadolescen* or juvenile*)) OR abstract((Adolescen* or Teen or Teens or Teenager* or Youth* or Elementary-school* or Middle-school* or High-school* or School-age* or Preteen or Preteens or Preteenager* or Prepub* or Puberty or Pubescen* or Preadolescen* or juvenile*)) |
| Databases: ProQuest Dissertations & Theses Global |
| Results: 264804 |
|  |
| Set#: S11 |
| Searched for: title((Young NEAR/2 (person* or people* or patient*)) ) OR abstract((Young NEAR/2 (person* or people* or patient*)) ) |
| Databases: ProQuest Dissertations & Theses Global |
| Results: 21273 |
|  |
| Set#: S12 |
| Searched for: [S10] OR [S11] |
| Databases: ProQuest Dissertations & Theses Global |
| These databases are searched for part of your query. |
| Results: 277023 |
|  |
| Set#: S13 |
| Searched for: [S4] AND [S8] AND [S12] |
| Databases: ProQuest Dissertations & Theses Global |
| These databases are searched for part of your query. |
| **Results: 56** |
